# The “Leicester, Leicestershire, and RUtland Chronic Kidney Disease Integrated Care Delivery Project” (LUCID) programme update: the impact on novel kidney therapies

**DOI:** 10.1093/ckj/sfaf312

**Published:** 2025-10-06

**Authors:** Rupert W Major, Niraj Lakhani, Nil Sanganee, Gillian Stead, Dipesh Patel, Matthew Graham-Brown, James O Burton

**Affiliations:** School of Medical Sciences – Public Health and Epidemiology Division, College of Life Sciences, University of Leicester, Leicester, UK; University of Hospitals of Leicester NHS Trust, Leicester, UK; Leicester, Leicestershire and RUtland Integrated Care Board, Leicester, UK; Willows Health Primary Care Network, Leicester, UK; Leicester, Leicestershire and RUtland Integrated Care Board, Leicester, UK; School of Medical Sciences – Division of Cardiovascular Sciences, College of Life Sciences, University of Leicester, Leicester, UK; Leicester, Leicestershire and RUtland Integrated Care Board, Leicester, UK; University of Hospitals of Leicester NHS Trust, Leicester, UK; NIHR Leicester Biomedical Research Centre, University of Leicester, Leicester, UK; University of Hospitals of Leicester NHS Trust, Leicester, UK; School of Medical Sciences – Division of Cardiovascular Sciences, College of Life Sciences, University of Leicester, Leicester, UK; NIHR Leicester Biomedical Research Centre, University of Leicester, Leicester, UK

To the Editor,

We previously reported the Leicester, Leicestershire, and RUtland Chronic Kidney Disease Integrated Care Delivery (LUCID) programme, which demonstrated an integrated care model between primary and secondary care for CKD in England [1]. We now provide an update focusing on medicines optimization, prescribing of novel therapies, and updated programme outcomes.

As of mid-2025, national uptake of finerenone remains low with marked inter-integrated care system (regional) variation despite robust evidence of cardiorenal benefit in patients with type 2 diabetes and CKD [2, 3]. Similar trends are seen with sodium zirconium cyclosilicate (SZC), where prescribing is increasing but remains modest relative to the estimated prevalence of hyperkalaemia in CKD and heart failure. Finerenone has been assessed as cost effective for patients with type 2 diabetes, CKD stage 3a–4, and albuminuria (A2–A3) where RAAS inhibitors and SGLT2 inhibitors are prescribed or not tolerated [3]. These prescribing patterns highlight a persistent implementation gap between evidence, NICE guidance, and clinical practice.

We analysed open-source prescribing data from OpenPrescribing.net [2]. The LUCID programme has embedded medicines optimization pathways for CKD within clinical workflows for multidisciplinary team meetings. All analysis was performed in Stata v.18.0 (code available on request). The number of people eligible for finerenone was estimated using national type 2 diabetes prevalence data combined with KDIGO 2024 estimates of CKD stage 3a–4 with A2–A3 albuminuria [4]. We fitted a multi-level mixed-effects regression model with finerenone prescribing rate per 100 eligible patients as the outcome. The model included LUCID activity status (active versus non-active), date of prescribing data (treated as the first day of each month), and their interaction, with GP practice list size (weighted for population size). Date for the model was restricted to after 15 May 2024, the date of local approval of finerenone. A value of *P* < .05 was treated as statistically significant.

## UPDATED PROGRAMME OUTCOMES

Up to 29 May 2025, 2816 clinical discussions about 1828 people living with CKD have taken place within LUCID. Co-morbidity burden remains high: 79.2% had hypertension, 54.2% diabetes, and 15.8% heart failure. A total of 1358 (48.2%) MDT episodes led to medicines optimization, while 296 (10.5%) and 228 (8.1%) resulted in expedited or avoided referrals, respectively.

## PRESCRIBING DATA

**Figure 1:**
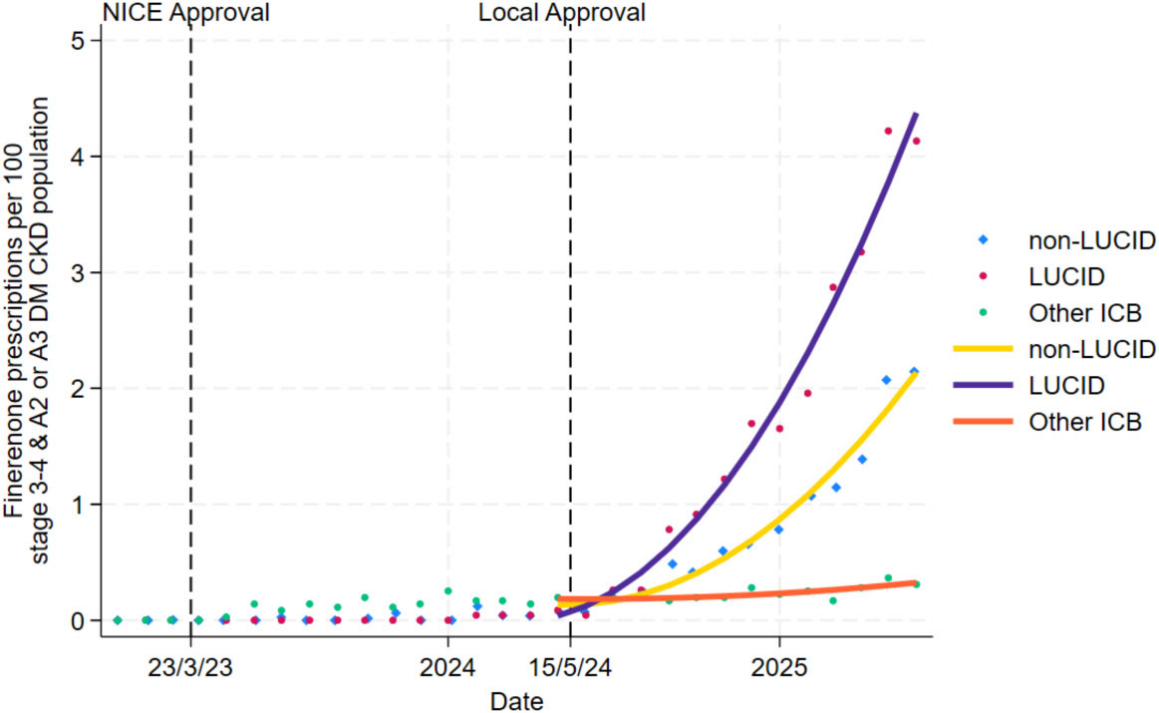
Prescribing rates of finerenone based on estimated eligible population size of T2DM CKD population with eGFR stages 3–4 and A2-3 albuminuria. Axes show prescribing rate per 100 eligible patients (*y*-axis) against time (*x*-axis). NICE Technology Appraisal approval was granted on 23 March 2023 with local system approval on 15 May 2024. Dots represent point estimates for prescribing. Lines represent fractional polynomial best fit weighted by population size for each point estimate. Purple, yellow, and orange lines are best fit for LUCID-active areas, non-LUCID-active areas in local system, and an anonymized comparator system (matched for prevalence of diabetes, urban/rural split, and deprivation).

National NICE guidance for finerenone was issued on 23 March 2023, with local approval in our integrated care system on 15 May 2024. Based on a reported prevalence within our system for type 2 diabetes of 83 050 and a 5% prevalence of CKD stage 3a–4 with A2–A3 albuminuria, we estimated 4153 people might be eligible for finerenone. In the following year after local approval, our clinical system had the highest rate of finerenone prescribing in England per estimated number of the eligible population [2]. Prescribing of finerenone was significantly higher in LUCID-active areas: 95 prescriptions (rate increase of 4.27/100 eligible/year) compared with 36 (1.96/100 eligible/year) in non-LUCID areas (*P* < .001). Figure 1 demonstrates this association. Similar but smaller trends were observed for SZC (58 vs 34 prescriptions, *P* < .001).

## DISCUSSION

Our updated findings confirm that people reviewed with the LUCID framework remain a high-risk, multimorbid population in whom the use of novel therapies remains limited. LUCID is an evidence-based, scalable, digital led framework focused on medicines optimization and risk stratification that can rapidly deliver cost-effective evidence-based medical therapies for people living with CKD. Barriers to uptake of novel therapies remain multi-factorial including: limited clinician awareness of new evidence-based medication, short-term cost pressures to systematic roll out and lack of systematic prescribing prompt implementation. LUCID has begun to address these issues through its education and system support mechanisms. Ongoing work will focus on barriers to uptake, including cost, clinician awareness, and integration into prescribing systems. Our analysis also demonstrates the value of open-source data for monitoring uptake, providing a scalable, minimal-burden approach for other health systems to perform similar analysis.

As both finerenone and SZC require secondary care initiation in our system, we did not undertake similar analyses for RAAS inhibitors, SGLT2 inhibitors, or lipid-lowering therapies. This approach also avoided bias from unmeasurable primary care prescribing variation. Our estimates therefore likely underestimate the overall impact of medicines optimization within LUCID. Approximately 130 episodes of optimization were related to finerenone or SZC out of ∼1300 in total, suggesting that these agents accounted for ∼10% of medicines optimization activity. This approach also avoided bias from unmeasurable primary care prescribing variation.

## CONCLUSION

Integration of prescribing data into LUCID highlights opportunities for medicines optimization and accelerated adoption of novel kidney therapies. The persistent gap between evidence and practice for finerenone and SZC underscores the importance of system-level strategies such as LUCID in achieving equitable and evidence-based kidney care.
